# Optomagnetic read-out enables easy, rapid, and cost-efficient qualitative biplex detection of bacterial DNA sequences

**DOI:** 10.1002/biot.201400615

**Published:** 2015-01-09

**Authors:** Rebecca S Bejhed, Teresa Zardán Gómez de la Torre, Peter Svedlindh, Mattias Strömberg

**Affiliations:** Department of Engineering Sciences, Uppsala University, Ångström LaboratoryUppsala, Sweden

**Keywords:** Biplex detection, Magnetic nanobeads, Optomagnetic setup, Padlock probe ligation, Rolling circle amplification

## Abstract

There is an increasing need to develop novel bioassay methods for low-cost, rapid, and easy-to-use multiplex detection of pathogens in various fields ranging from human infectious disease diagnosis, drinking water quality control, to food safety applications. Due to their unique advantages, magnetic and optomagnetic bioassay principles are particularly promising for biodetection platforms that will be used in developing countries. In this paper, an optomagnetic method for rapid and cost-efficient qualitative biplex detection of bacterial DNA sequences is demonstrated. Within less than two hours, the assay gives an answer to whether none, both, or only one of the bacterial DNA sequences is present in the sample. The assay relies on hybridization of oligonucleotide-functionalized magnetic nanobeads of two different sizes to rolling circle amplification (RCA) products originating from two different bacterial targets. The different bead sizes are equipped with different oligonucleotide probes, complementary to only one of the RCA products, and the read-out is carried out in the same sample volume. In an optomagnetic setup, the frequency modulation of transmitted laser light in response to an applied AC magnetic field is measured. The presented methodology is potentially interesting for low-cost screening of pathogens relating to both human and veterinary medicine in resource-poor regions of the world.

## 1 Introduction

Novel bioassay methods for low-cost, rapid, and easy-to-use multiplex detection of pathogens are increasingly demanded in diverse fields ranging from human infectious disease diagnostics [1, 2], drinking water quality control [3–5] to food safety applications [6–8]. Most of today's commercial instruments for pathogenic target deoxyribonucleic acid (DNA) detection rely on fluorescence read-out, often in combination with target amplification through PCR. This enables a high degree of multiplexing and excellent sensitivity, however, at the expense of a high risk of false positives, relatively costly equipment, and the need for specially trained personnel. In contrast, magnetic [9, 10] and optomagnetic bioassay principles [11–13] offer unique advantages in terms of very low background signals and associated low-cost equipment and are therefore promising for biodetection platforms to be used in developing countries [14]. Particularly relevant applications could, for instance, be in-field, rapid and cost-efficient qualitative multiplex monitoring of pathogens. If one or more positive answers are obtained, samples could be sent to a central laboratory for further analysis, such as identification of strains, etc.

In this paper we present an optomagnetic method for rapid and cost-efficient qualitative biplex detection of bacterial DNA sequences. Within less than two hours, the assay gives a qualitative answer to whether none, both, or only one of the bacterial DNA sequences is present in a sample. The assay begins with a padlock probe ligation and rolling circle amplification (RCA) protocol (about 70 min) for highly specific target recognition and isothermal enzymatic amplification. The resulting two types of RCA products in the form of macromolecular coils of ssDNA (here denoted DNA coils) with a repeating sequence motif are mixed with magnetic beads (exhibiting Brownian relaxation behavior) of two different sizes, each functionalized with a detection oligonucleotide complementary to only one type of DNA coil. It should be noted that the padlock probe ligation reaction constitutes highly specific target recognition since both the 5' and 3' ends of the linear padlock probe are designed to bp next to each other on the target strand. After an incubation step of 20 min and transfer to a disposable cuvette, the coil-bead sample is measured using an optomagnetic system (measurement time about a few minutes) in which the sample, while being subjected to an alternating current (AC) magnetic field (perpendicular to the optical path), is illuminated by a laser beam and the transmitted light is collected by a photodetector. The field-induced sample response is measured as the second harmonic component, *V*_2_ = *V*_2_' + *V*_2_'', of the output voltage from the photodetector, normalized with respect to the total intensity of transmitted light, *V*_0_. We consider two data analysis approaches; firstly *V*_2_'/*V*_0_ and secondly the phase angle response, *ζ* = arctan (*V*_2_'/*V*_2_''), vs. frequency of the AC magnetic field. Beads bound to DNA coils will respond differently than free beads, which will be reflected in the shape of the curves. We are able to show that each combination of presence/absence of the two targets has a distinct phase angle signature, thereby enabling a straightforward qualitative biplex read-out. It should be noted that in previous work [15] we demonstrated biplex detection of bacterial DNA sequences using a commercial and portable AC susceptometer device (DynoMag) for read-out in terms of measuring the AC magnetization of the sample. In the current work we have achieved major advancements in terms of performing the read-out in a device having considerable potential to be made at a much lower cost than the DynoMag system (about a factor of 100 times lower). Furthermore, the optomagnetic system is considerably easier to miniaturize and is also much more compatible with microfluidics for automated sample preparation.

## 2 Materials and methods

Sequences of targets, padlock probes, and detection oligonucleotides can be found in Supporting information, Table S1.

### 2.1 Conjugation of detection oligonucleotides to magnetic nanobeads

Two 200-μL batches of oligonucleotide-functionalized magnetic beads were prepared according to protocols described in detail in Supporting information, Section S1. Detection oligonucleotide for *Escherichia coli* (EC) was conjugated to 250-nm beads (nanomag-D avidin, Micromod), and detection oligonucleotide for *Vibrio cholerae* (VC) was conjugated to 100-nm beads (Brownian nanofluid (BNF)-Starch avidin, Micromod, Germany). The two bead batches were further diluted with PBS to a final concentration of 400 μg/mL, after which they were mixed at a volume to volume ratio of 1:2 to get final approximate concentrations of 130 and 270 μg/mL of 250- and 100-nm beads, respectively.

### 2.2 Padlock probe target recognition, ligation, and rolling circle amplification

Target recognition and RCA were performed essentially as previously described in ref. [15], for details see Supporting information, Section S2.

### 2.3 Optomagnetic setup

The optomagnetic setup comprises a Blu-ray laser source, a photodetector, a pair of electromagnets, a sample holder, a current source, a data acquisition (DAQ) unit, and a computer; for details, see Supporting information, Section S3. A schematic illustration of the setup is shown in Supporting information, Fig. S1. An AC magnetic field is applied using the electromagnets, and the frequency modulation of the photodetector voltage signal is measured using a lock-in amplifier with the magnetic field frequency as the reference. The magnetic beads respond to the applied AC field by the formation and disruption of chain-like superstructures. The modulation of transmitted light by this chain formation/disruption dynamics is measured as the second harmonic component of the photodetector voltage signal, divided into its in- and out-of-phase components. Theory behind the optomagnetic measurement principle can be found in Supporting information Section S4 as well as in ref. [16]. In particular, Supporting information, Fig. S2 shows the relation between the AC magnetic and the optomagnetic sample response.

### 2.4 Sample preparation and read-out

Fifteen μL of bead solution was gently mixed with 15 μL of DNA coil solution of different compositions and concentrations. The solution was incubated for 20 min at 55 °C, after which it was diluted with 30 μL of a 50–50 buffer mixture (50 v/v % of 1 × PBS pH 7.4 and 50 v/v % of hybridization buffer). The final solution was transferred to a cuvette and measured using the optomagnetic setup in the frequency range 1–200 Hz with an AC magnetic field amplitude of 2.58 mT.

## 3 Results and discussion

**Figure 1:**
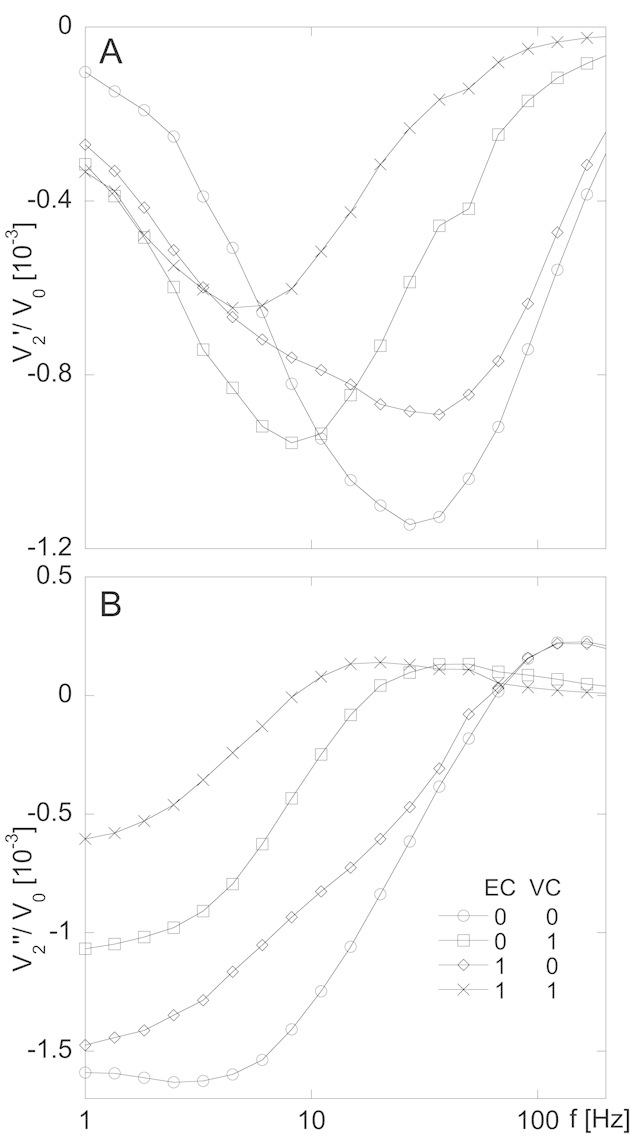
Biplex detection of EC and VC DNA coils, generated through target recognition by padlock probe ligation followed by RCA (see protocols in Supporting information Section S2), using 250- and 100-nm magnetic beads functionalized with detection oligonucleotides for EC and VC, respectively (see protocol in Supporting information, Section S1). Fifteen μL of DNA coil solution (both EC and VC) and 15 μL of bead suspension (mixture of the two sizes) were incubated for 20 min at 55 °C and diluted with 30 μL of a buffer mixture prior to measurements in an optomagnetic system. In this setup the sample contained in a disposable cuvette, while being subjected to an AC magnetic excitation field (perpendicular to the optical path), is illuminated by a laser beam and the transmitted light is collected by a photodetector. Four combinations of DNA coil concentrations were measured upon; 0–0, 0–1, 1–0, and 1–1, where the first figure represents the concentration of EC coils and the second figure represents the concentration of VC coils in nM. The second harmonic component, *V*_2_ = *V*_2_' + *V*_2_'', of the photodetector voltage output signal was measured as a function of frequency of the applied magnetic excitation field. Panel A shows the normalized in-phase component, *V*_2_'/*V*_0_, and panel B show the normalized out-of-phase component, *V*_2_''/*V*_0_, where *V*_0_ is the total intensity of transmitted light. The curves are based on the average of triplicate measurements.

Panels A and B in Fig. 1 show in- (*V*_2_'/*V*_0_) and out-of-phase (*V*_2_''/*V*_0_) vs. frequency spectra for four selected DNA coil concentration combinations, respectively. It can be observed that all four curves have clearly distinct signatures, different in shape and/or peak frequency. For no DNA coils present (EC 0, VC 0) a comparably narrow *V*_2_' peak is seen at a frequency corresponding to one in-between the two individual free-bead peaks, which could be considered as the sum of the peaks for 250- and 100-nm beads. When measuring on samples with only 250-nm beads and EC-coils (see Supporting information, Fig. S3A), the free-bead peak is located at 15 Hz, whereas the free-bead peak for 100-nm beads (see Supporting information, Fig. S3B) is located at 40 Hz. It is reasonable to assume that the result from measuring on a sample containing both bead sizes would be represented by a peak at a frequency somewhere in between those two values. When DNA coils in high enough concentration are present, the free-bead peak is suppressed in favor of the formation of a new bound-bead peak at a lower frequency. This *V*_2_' peak is tentatively explained by the formation of chains by beads bound in DNA coils. As can be seen in Supporting information, Fig. S3A and B, the bound-bead peak for 250-nm beads in EC coils appears at 5 Hz whereas the bound-bead peak for 100-nm beads in VC coils appears at a slightly higher frequency. Using this information, the interpretation of the curve shapes and peak frequencies of Fig. 1B is straightforward. For the sample EC 1, VC 1, the peak represents the sum of the two bound-bead peaks and is thus located at 5 Hz. For sample EC 1, VC 0, the measured *V*_2_' spectrum represents the sum of the contributions from EC coils with bound 250-nm beads and free 100-nm beads. Accordingly, the curve shows one peak at 37 Hz representing the free 100-nm beads and a signature of one low-frequency peak representing the response from EC coils with bound 250-nm beads. For the sample EC 0, VC 1, the *V*_2_' curve is a sum of the response from free 250-nm beads and VC coils with bound 100-nm beads. Since these two peaks overlap much more than for the case of the EC 1, VC 0 sample, the resulting curve is much narrower. In the EC 0, VC 1 curve, a small residual peak at 50 Hz can be seen, corresponding to the response from a small amount of free 100-nm beads. The explanation for the residual free 100-nm bead peak being located at a somewhat higher frequency compared to the measured free-bead peak at 40 Hz could be explained in terms of the existence of a small part of the bead size distribution (corresponding to beads with the smallest sizes) having few or no oligonucleotides, therefore unable to bind to DNA coils.

**Figure 2:**
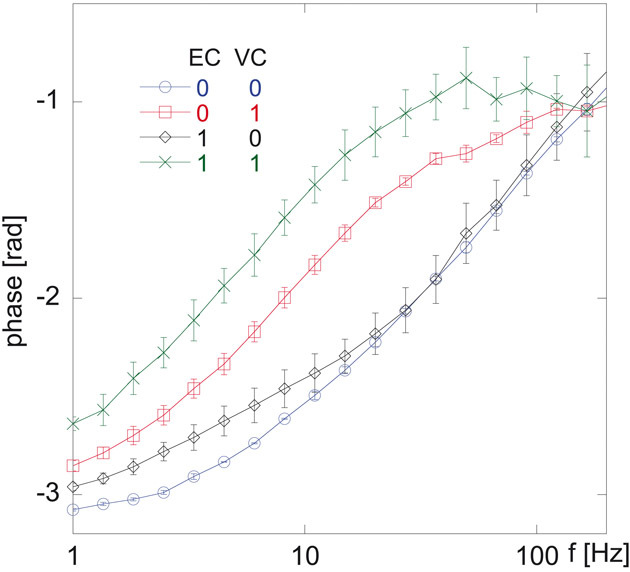
Biplex detection of EC and VC DNA coils using 250- and 100-nm magnetic beads functionalized with EC and VC detection probes, respectively, according to Supporting information, Section S1. The DNA coils were generated according to protocols given in Supporting information, Section S2. Fifteen μL of DNA coil solution (both EC and VC) and 15 μL of beads (mixture of the two sizes) were incubated for 20 min at 55 °C and diluted with 30 μL of a buffer mixture prior to measurements in an optomagnetic system. The figure shows phase angle vs. frequency spectra for four different combinations of DNA coil concentrations; 0–0, 0–1, 1–0, 1–1 (in nM), where the first figure represents EC and the second figure represents VC. The phase angle is defined as *ζ* = arctan (*V*_2_'/*V*_2_'') where *V*_2_' and *V*_2_'' is the in-phase and out-of-phase components of the second harmonic of the photodetector voltage output signal. Each curve is based on triplicate measurements with error bars corresponding to one SD.

In recent studies [16] we have demonstrated that the phase angle analysis method is superior to the turn-off approach (decrease of *V*_2_' peak amplitude upon increasing DNA coil concentration) with regards to SDs of the measured samples. Thus, we proceeded by considering the phase angle *ζ* = arctan (*V*_2_'/*V*_2_'') vs. frequency spectrum. In Fig. 2, the *ζ* vs. frequency spectra belonging to the four samples in Fig. 1 are shown (see Supporting information, Fig. S4C for all samples). With this analysis method the four concentrations result in curves with clearly distinct signatures. Additionally it should be noted that, according to Supporting information, Fig. S4C, a DNA coil concentration of 0.5 nM gives the same response as obtained for the 1-nM coil concentration; an observation that further confirms the qualitative assay characteristics of our method. Important to emphasize is that in ref. [16] (Supporting information, Fig. S7) we have shown using 250-nm beads that the phase angle spectrum of a negative control sample does not significantly differ from that of a sample containing 500 pM of DNA coils non-complementary to the beads. This implies that in the biplex assay the binding of beads to complementary DNA coils is not significantly influenced by the presence of non-complementary DNA coils.

## 4 Concluding remarks

We have demonstrated qualitative biplex detection of *E. coli* and *V. cholerae* DNA sequences in an optomagnetic measurement setup. Within less than two hours an answer can be obtained. The presented methodology is potentially interesting for low-cost screening of pathogens relating to both human and veterinary medicine in resource-poor regions of the world.

## Abbreviations

AC: alternating current
DAQ: data acquisition unit
DNA: deoxyribonucleic acid
EC: *Escherichia coli*
RCA: rolling circle amplification
VC: *Vibrio cholerae*

## Notes

Supporting information available online
