# Supplementary material for: Optomagnetic read-out enables easy, rapid, and cost-efficient qualitative biplex detection of bacterial DNA sequences
Source: Biotechnol J. 2015 Jan 9;10(3):469–72. doi: 10.1002/biot.201400615 (PMC4406137; doi:10.1002/biot.201400615)
Supplement: Supplementary file 1 [file biot0010-0469-sd1.pdf]

Biotechnology
Journal

Supporting Information for DOI 10.1002/biot.201400615

Optomagnetic read-out enables easy, rapid and cost-efficient qualitative biplex detection of bacterial DNA sequences

Rebecca S. Bejhed, Teresa Zardán Gómez de la Torre, Peter Svedlindh and Mattias Strömberg

© 2014 Wiley-VCH Verlag GmbH & Co. KGaA, Weinheim

Supporting information for

Biotech Method

Optomagnetic read-out enables easy, rapid and cost-efficient qualitative biplex detection of bacterial DNA sequences

Rebecca S. Bejhed, Teresa Zardán Gómez de la Torre, Peter Svedlindh and Mattias Strömberg

Department of Engineering Sciences, Uppsala University, Ångström Laboratory, Uppsala, Sweden

Correspondence: Dr. Mattias Strömberg, Department of Engineering Sciences, Uppsala University, The Ångström Laboratory, Box 534, SE-751 21 Uppsala, Sweden

E-mail: mattias.stromberg@angstrom.uu.se

Table S1. Sequences of targets, padlock probes and detection oligonucleotides. Parts of the padlock probes matching the VC/EC detection oligo are highlighted in red.

**Name**	**Sequence**
Vibrio cholerae target (VC)	5'-CCCTGGGCTCAACCTAGGAATCGCATTTG-3'
Escherichia coli target (EC)	5'-ACGTCGCAAGACCAAAGAGGGGGACCT-3'
Padlock probe for VC	5'-TAGGTTGAGCCCAGGGACTTCTAGAGTGTACCGACCTCAGTAGCCGTGACTATCGACTTGTTGATGTCATGTGTCGCACCAAATGCGATTCC-3'
Padlock probe for EC	5'-CTTTGGTCTTGCGACGTCAGTGGATAGTGTCTTACACGATTTAGAGTGTACCGACCTCAGTAGCCGTGACTATCGACTAGGTCCCCCT-3'
Detection oligo for VC	Biotin-5'-TTTTTGTTGATGTCATGTGTCGCAC-3'-Fam
Detection oligo for EC	Biotin-5'-TTTTTTTTTTTTTTTTTTTTGTGGATAGTGTCTTACACGA-3'-Fam

S1: Conjugation of detection probes to magnetic nanobeads

250 nm beads (nanomag-D avidin, Micromod) were conjugated with a 1 000-fold oligonucleotide excess. 40 µl of magnetic bead suspension (10 mg/ml of solid content, 4.9×10^{11} beads/ml) were washed twice with 1×Wtw buffer (10 mM Tris-HCl [Sigma-Aldrich, USA], 5 mM EDTA [Sigma-Aldrich], 0.1% Tween20 [Sigma-Aldrich], 0.1 M NaCl [Sigma-Aldrich]) using a magnetic separation stand and re-suspended in 50 µl of 1×Wtw buffer. 3.25 µl of 10 µM EC detection oligonucleotide solution (Biomers, Germany) was added to the magnetic beads. The bead-oligo solution was incubated for 15 minutes at room temperature while shaking the tube after which it was washed twice with 1×PBS pH 7.5 (Sigma-Aldrich) and re-suspended in 200 µl of PBS (~2 mg/ml of beads). The final solution was transferred into a sealed glass vial and stored in fridge at 4°C. A batch of oligonucleotide-conjugated 100 nm beads (BNF-Starch avidin, Micromod, Germany) with 100-fold oligonucleotide excess was prepared following the same protocol as for the 250 nm beads except for starting with 40 µl of magnetic bead suspension (10 mg/ml of solid content, 6×10^{12} beads/ml) and adding 4.15 µl of 10 µM VC detection oligonucleotide solution to the magnetic beads.

S2: Padlock probe target recognition, ligation and rolling circle amplification

In summary, 6 µl of either VC or EC synthetic DNA template was added to a 99 µl ligation mixture consisting of 10 x Φ29 buffer, 20mM ATP, 1 µM phosphorylated VC or EC padlock probe, 1 U/µl T4 DNA ligase and MQ water. Hybridization and ligation was performed at 37^0C during 15 min. Desired amounts of VC and EC ligation mixes were added (see Table S2) to a RCA mixture containing 10 x Φ29 buffer, 2.5 mM dNTP, 2 µg/µl BSA, Φ29 DNA polymerase and MQ water. RCA was performed at 37^0C during 60 min followed by enzyme activation at 65^0C for 5 min. Final DNA coil solutions were obtained by adding hybridization buffer consisting of 1 M Tris-HCl, 0.5 M EDTA, 10% Tween-20, 5 M NaCl and MQ water.

Table S2. Preparation of DNA coils for biplex detection.

**VC DNA coil conc. (nM)**	Vol. of VC lig. mix 2 nM (µl)	**EC DNA-coil conc. (nM)**	Vol. of EC lig. mix 2 nM (µl)
0	0	0	0
0.5	10	0.5	10
0.5	10	0	0
0	0	0.5	10
1	20	1	20
1	20	0	0
0	0	1	20

S3: Optomagnetic system

The set-up used for measuring the optomagnetic effect is based on an unfocused 405 nm Blu-ray laser source (Sony Blu-ray optical unit, Sony, JP) and a photodetector (PDA36A, Thorlabs Inc., U.S.A.), see Figure S1. The laser source, which is powered by a current source and computer software controlled, provides a linearly polarized light beam with a beam diameter of 2 mm. An optically transparent cuvette (Sarstedt AG & Co, Nümbrecht, Germany) centered between a pair of electromagnets (1433428C, Murata Power Solutions Inc., U.S.A.) was positioned in the beam path. The distance between the electromagnets was 20 mm, and the distance between laser source and detector was 115 mm. At the bottom of the cuvette there is a 2 mm wide, 10 mm long trench where the liquid sample is located. Since the cuvette is oriented with the short side of the trench towards the laser, the optical probing volume is estimated to approximately 30 mm^3. In previous studies, the trench has been orientated with its long side towards the laser, i.e., using a smaller probing volume. The electromagnets were powered by an AC current source and computer controlled through Labview. The maximum AC magnetic field amplitude, measured with a Hall sensor (HGT-1020, Lake Shore Cryotronics Inc., U.S.A.) was restricted to about 2.6 mT in the current set-up. To ensure constant field amplitude and phase at all frequencies, the self-inductance of the electromagnets was corrected for. The laser beam was aimed at the bottom of the cuvette, and the transmitted light was recorded by the photodetector. The AC magnetic field was applied perpendicular to the laser beam for all measurements described in this paper. To ensure no interference from other light sources, the laser, electromagnets, cuvette, and detector were covered during measurements. Furthermore, the optical table with the laser, photodetector and central section with electromagnets and cuvette holder was placed on a heavy concrete fundament in order to reduce measurement noise coming from vibrations. A data acquisition unit (NI USB-6341, National Instruments, U.S.A.) converted the detector signal from analogue to digital, after which it was further treated in the computer by a FFT enabled lock-in function.

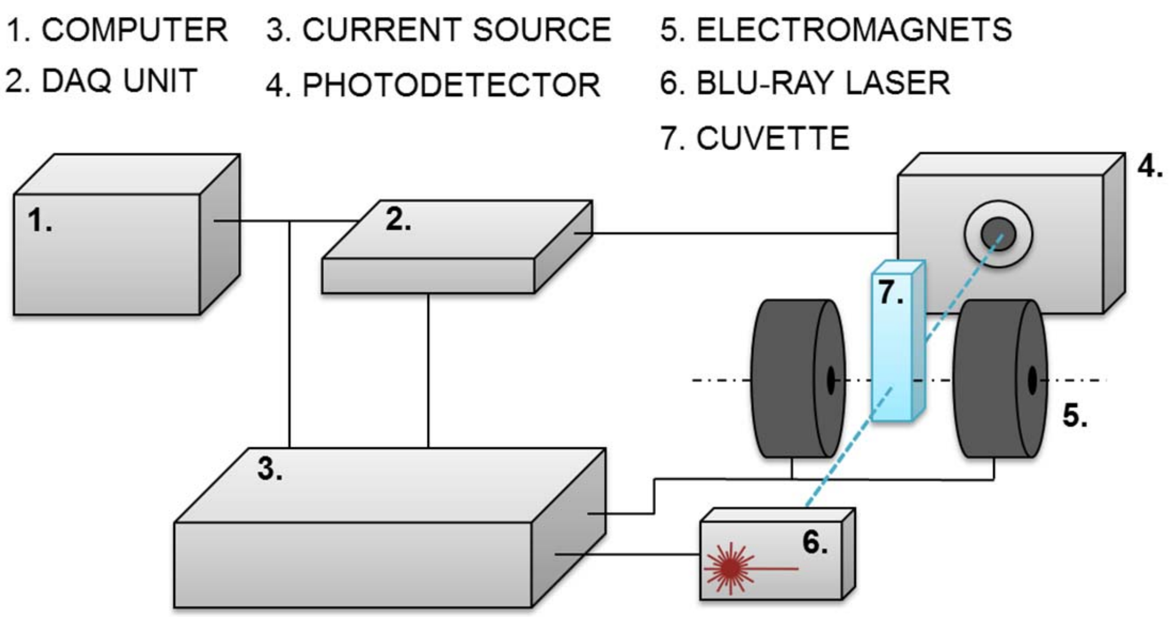

Figure S1. Schematic illustration of the optomagnetic set-up. The liquid sample, contained in an optically transparent cuvette (7), is placed between two identical electromagnets (5). A Blu-ray laser source (6) generates a laser beam aimed at the bottom of the cuvette. The transmitted light detected by a photo detector (4) is recorded vs. time using a DAQ unit (2). The laser and electromagnets are powered by a current source (3). A computer (1) controls the entire set-up and performs the software based lock-in detection.

S4: Optomagnetic measurement principle

The optomagnetic measurement principle is based on the rotational dynamics of magnetic nanobeads. The magnetic nanobeads employed in this study have a remanent magnetic moment. This implies that the dominating relaxation mechanism upon a reversal of the magnetic field direction is a physical rotation of the particle, which is also known as Brownian relaxation. The characteristic frequency for Brownian relaxation dynamics is

$$f_B = \frac{k_B T}{6\pi\eta V_h}, \quad (1)$$

where k_BT is the thermal energy, η is the dynamic viscosity and V_h is the hydrodynamic volume of the relaxing entity (e.g., a single magnetic nanobead). The dynamic magnetic behaviour can be described in term of the magnetic susceptibility χ with real and imaginary parts χ' and χ'', respectively. At low frequencies the magnetic nanobeads are able to rotate and follow the magnetic field, and the response is in-phase with the applied field. Therefore χ' is maximal. At higher frequencies the rotation of the magnetic nanobeads starts to lag behind the applied field. This leads to an increase in the out-of-phase component χ'' and a corresponding decrease of the in-phase component. The out-of-phase component χ'' attains its maximum value at the Brownian relaxation frequency f_B. A plot of the calculated magnetic susceptibility vs. frequency is shown in Fig. S2a.

When an AC sinusoidal magnetic field is applied at a frequency *f*, the magnetic nanobeads rotate physically to follow the magnetic field orientation and may form chain-like structures.

Upon reversal of the magnetic field direction, chains of magnetic nanobeads will tend to break up due to thermal agitation and because a magnetic torque is acting on the individual magnetic nanobeads. The rotation of individual beads (which are irregularly shaped) as well as the formation/disruption of chains of magnetic nanobeads will contribute to a field-induced modulation of the transmitted light intensity. In both cases, the dynamics is determined by the rotational behaviour of the individual beads, which follows the Brownian relaxation dynamics. Magnetic nanobeads bound to DNA coils may still give rise to a field-induced modulation of the transmitted intensity, but due to the larger size of the bead-coil cluster this takes place at a lower characteristic frequency. Moreover, the size increase and possibility of several beads bound to the same DNA coil may also change the scattering properties of the cluster. As the optomagnetic signal is insensitive to the sign of the magnetic field, it can be shown that the features of the out-of-phase magnetic response are mainly observed in the in-phase 2nd harmonic optomagnetic signal. In Donolato et al 2014, we have presented a simple analytical model linking the 2nd harmonic optomagnetic spectra to the magnetic susceptibility spectra of magnetic nanobeads,

$$\begin{aligned} V_2' &= -\tfrac{1}{\sqrt{2}} V_{AC} (\tilde{\chi}')(\tilde{\chi}'') \\ V_2'' &= -\tfrac{1}{2\sqrt{2}} V_{AC} \left[(\tilde{\chi}')^2 - (\tilde{\chi}'')^2 \right] \end{aligned} \qquad (2)$$

where V_{AC} is positive if the optical transmission is higher when the AC magnetic field is large compared to when it is small, and negative otherwise, and the tilde's on the χ's indicate that they are normalized with the low-frequency susceptibility. The sign of V_{AC} depends on the optical scattering properties and the measurement geometry. For a geometry where the transmission is measured perpendicular to the axis of the applied magnetic field, as used in the present study, it is generally found that V_{AC} is negative for nanobeads with sizes smaller than about 130 nm for blue laser light (λ = 405 nm). When the size increases beyond this value, oscillations in V_{AC} are observed such that V_{AC} first becomes positive (e.g., for 250 nm magnetic nanobeads) and then negative for even larger scattering entities. This originates from the oscillation of the scattering cross-section with particle size as can be accounted for by Mie scattering theory. The detailed interplay between the scattering properties and physical arrangement of particles when bound to DNA coils is still under investigation.

Fig. S2 shows magnetic susceptibility spectra calculated from the Debye/Cole-Cole model (Cole and Cole 1941) (with Cole-Cole parameter α = 0) and the corresponding optomagnetic spectra calculated using Eq. (2). The peak frequency observed in the in-phase 2nd harmonic optomagnetic signal is closely related (although not identical) to f_B.

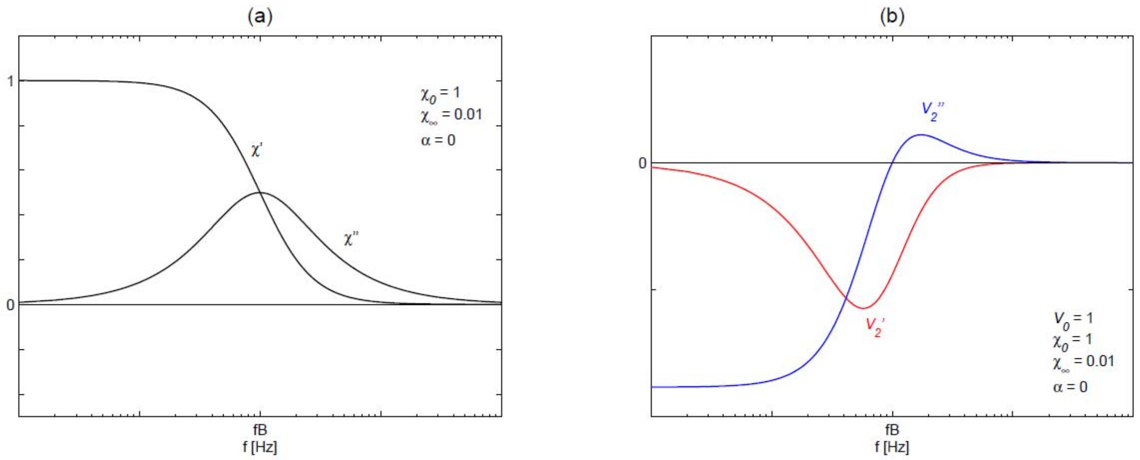

Figure S2. (a) Plots of the in- and out-of-phase components of the complex magnetic susceptibility (χ' and χ''). The peak of χ'' is located at the Brownian relaxation frequency f_B. (b) Plots of the calculated in- and out-of-phase components of the 2nd harmonic signal (calculated assuming a positive value of V_{AC}).

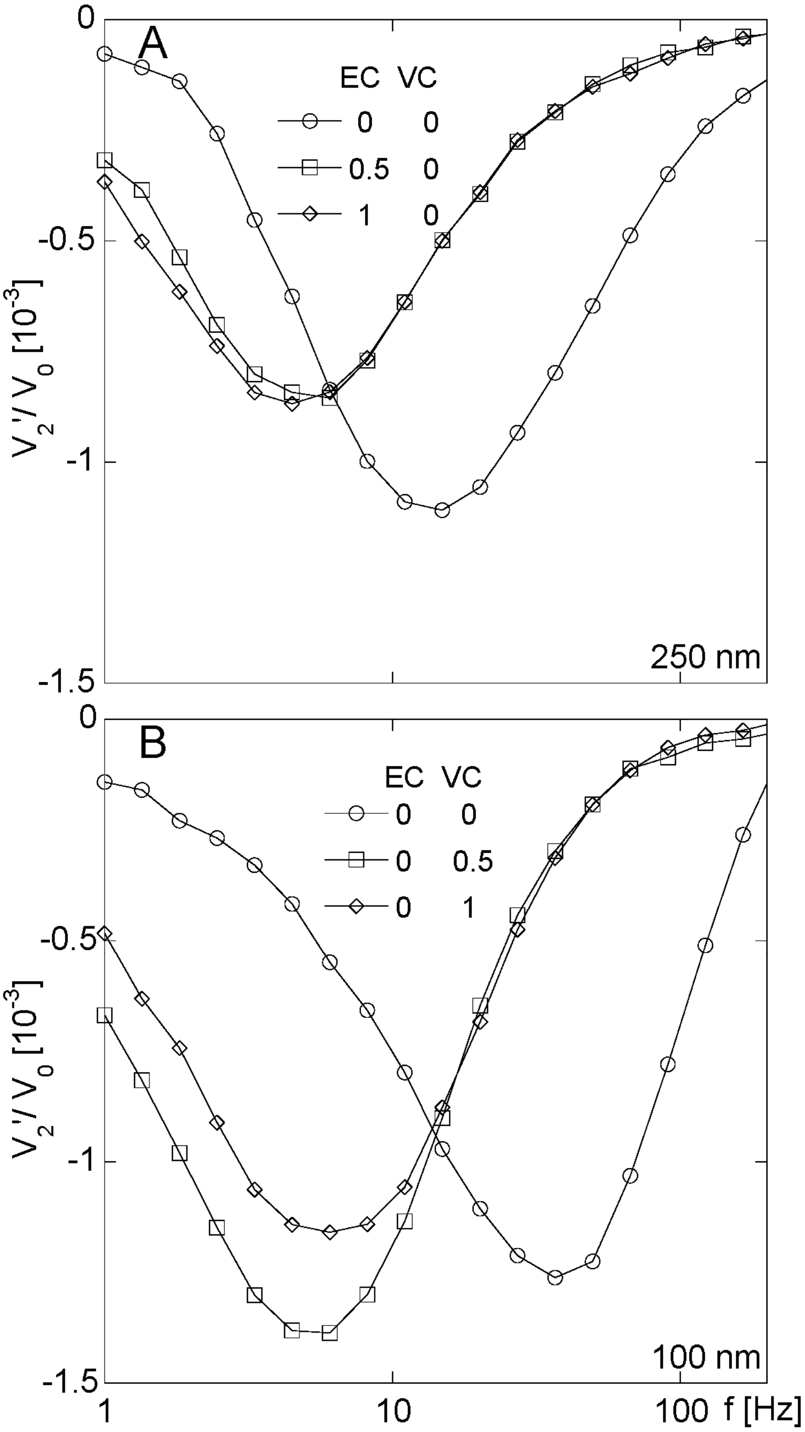

Figure S3. EC and VC DNA coils, generated using the padlock probe ligation and RCA protocols in section S2, has been detected using either 250 nm or 100 nm magnetic beads functionalized with EC and VC detection probes, respectively, according to section S1. 15 µl of DNA coil solution (both EC and VC coils) and either 5 µl 250 nm beads + 10 µl PBS or 10 µl 100 nm beads + 5 µl PBS were incubated for 20 minutes at 55°C and diluted with 30 µl of a buffer mixture prior to measurements.

Three combinations of DNA coil concentrations were measured upon for each bead size, where the first figure represents the concentration of EC coils and the second figure represents the concentration of VC coils, in nM. The second harmonic component, $V_2 = V_2' + iV_2''$, of the photodetector voltage output signal was measured as a function of the applied magnetic excitation field frequency. Normalized in-phase (V_2'/V_0) vs. frequency spectra for the different DNA coil concentrations using 250 nm beads (A) and 100 nm beads (B) has been plotted.

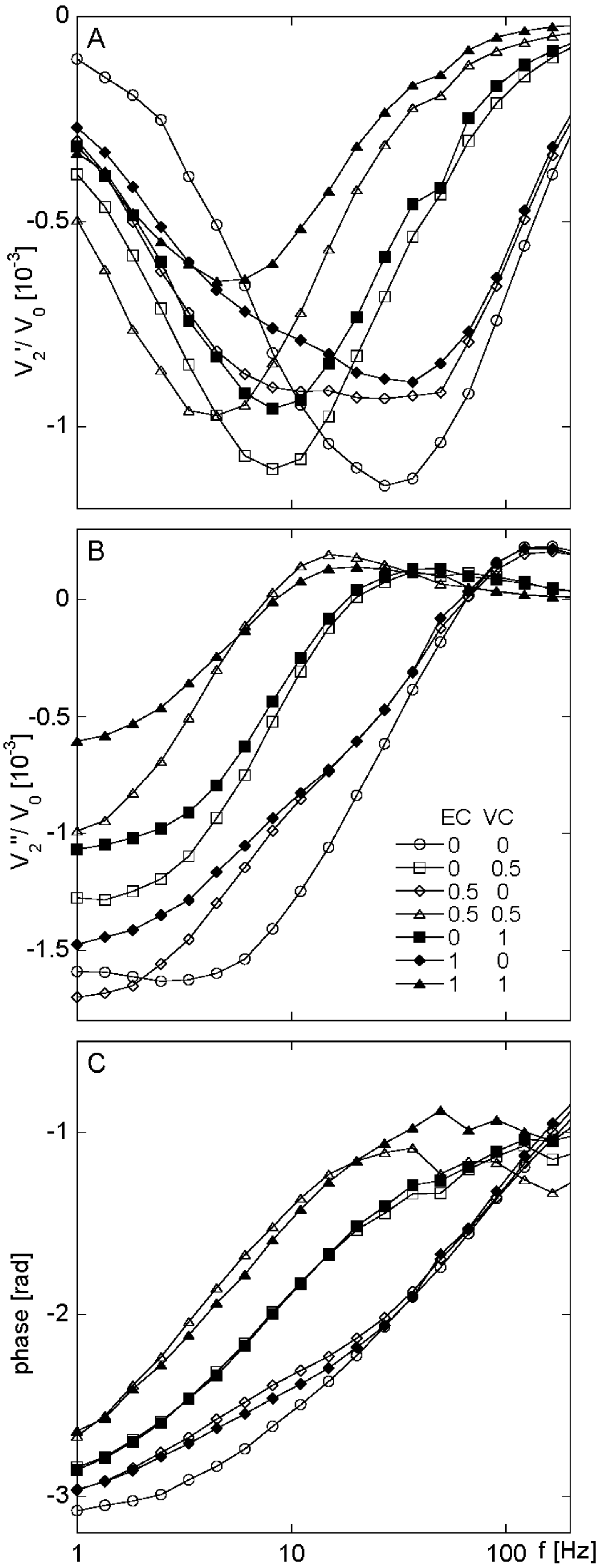

Figure S4. EC and VC DNA coils, generated using the padlock probe ligation and RCA protocols in section S2, has been simultaneously detected using 250 nm and 100 nm magnetic beads functionalized with EC and VC detection probes, respectively, according to section S1. 15 µl of DNA coil solution (both EC and VC) and 15 µl of bead suspension (mixture of both sizes) were incubated for 20 minutes at 55°C and diluted with 30 µl of a buffer mixture prior to measurements. Seven combinations of DNA coil concentrations were measured upon where the first figure represents the concentration of EC coils and the second figure represents the concentration of VC coils, in nM. The second harmonic component, $V_2 = V_2' + iV_2''$, of the photodetector voltage output signal was measured as a function of the frequency of the applied magnetic AC excitation field. Normalized in-phase (V_2'/V_0) (A), out-of-phase (V_2''/V_0) (B) and phase angle, $\xi = \arctan(V_2'/V_2'')$, vs. frequency spectra (C) are displayed. Each curve is based on the average of triplicates.

References

Cole, K.S., Cole, R.H., 1941. J Chem Phys 9, 341-351.

Donolato, M., Antunes, P., Bejhed, R.S., Zardán Gómez de la Torre, T., Østerberg, F.W., Strömberg, M., Nilsson, M., Strømme, M., Svedlindh, P., Hansen, M.F., Vavassori, P., 2014. *Submitted to Anal. Chem.*
